# Network Pharmacological Analysis of the Red Sea Sponge *Hyrtios erectus* Extract to Reveal Anticancer Efficacy of Corresponding Loaded Niosomes

**DOI:** 10.3390/md20100628

**Published:** 2022-10-01

**Authors:** Heba A. Abou-Taleb, Ahmed M. Sayed, Hesham Refaat, Faisal Alsenani, Eman Alaaeldin, Fatma A. Mokhtar, Usama Ramadan Abdelmohsen, Nourhan Hisham Shady

**Affiliations:** 1 Department of Pharmaceutics and Industrial Pharmacy, Merit University (MUE), New Sohag 82755, Egypt; 2 Department of Pharmacognosy, Faculty of Pharmacy, Al-Maaqal University, Basra 61014, Iraq; 3 Department of Pharmacognosy, Faculty of Pharmacy, Nahda University, Beni-Suef 62513, Egypt; 4 Department of Pharmaceutics, Faculty of Pharmacy, Minia University, Minia 61519, Egypt; 5 Department of Pharmaceutics, Faculty of Pharmacy, Deraya University, 7 Universities Zone, New Minia 61111, Egypt; 6 Department of Pharmacognosy, College of Pharmacy, Umm Al-Qura University, Makkah 21955, Saudi Arabia; 7 Department of Pharmacognosy, Faculty of Pharmacy, Alsalam University, Kafr El Zayat 31616, Egypt; 8 Department of Pharmacognosy, Faculty of Pharmacy, Minia University, Minia 61519, Egypt; 9 Department of Pharmacognosy, Faculty of Pharmacy, Deraya University, Universities Zone, Minia 61111, Egypt

**Keywords:** *Hyrtios*, marine sponge, metabolic, cytotoxicity, niosomes, network pharmacology, enrichment analysis

## Abstract

In this study, the LC-HRMS-assisted chemical profiling of *Hyrtios erectus* sponge led to the annotation of eleven major compounds (**1**–**11**). *H. erectus*-derived crude extract (HE) was tested in vitro for its antiproliferative activity against three human cancer cell lines, Hep-G2 (human liver cancer cell line), MCF-7 (breast cancer cell line), and Caco-2 (colon cancer cell line), before and after encapsulation within niosomes. *Hyrtios erectus extract* showed moderate in vitro antiproliferative activities towards the studied cell lines with IC_50_ values 18.5 ± 0.08, 15.2 ± 0.11, and 13.4 ± 0.12, respectively. The formulated extract-containing niosomes (size 142.3 ± 10.3 nm, PDI 0.279, and zeta potential 22.8 ± 1.6) increased the in vitro antiproliferative activity of the entrapped extract significantly (IC_50_ 8.5 ± 0.04, 4.1 ± 0.07, and 3.4 ± 0.05, respectively). A subsequent computational chemical study was performed to build a sponge–metabolite–targets–cancer diseases network, by focusing on targets that possess anticancer activity toward the three cancer types: breast, colon, and liver. Pubchem, BindingDB, and DisGenet databases were used to build the network. Shinygo and KEGG databases in addition to FunRich software were used for gene ontology and functional analysis. The computational analysis linked the metabolites to 200 genes among which 147 genes related to cancer and only 64 genes are intersected in the three cancer types. The study proved that the co-occurrence of compounds **1**, **2**, **3**, **7**, **8**, and **10** are the most probable compounds possessing cytotoxic activity due to large number of connections to the intersected cytotoxic genes with edges range from 9-14. The targets possess the anticancer effect through Pathways in cancer, Endocrine resistance and Proteoglycans in cancer as mentioned by KEGG and ShinyGo 7.1 databases. This study introduces niosomes as a promising strategy to promote the cytotoxic potential of *H.* erectus extract.

## 1. Introduction

The second cause of death all over the world after ischemic heart disease is cancer [1]. Cancer rates are predicted to elevate from 6.1 million cases to 10.6 million by 2030. [2]. Recently, natural metabolites possess a critical role in reducing the risk of cancer diseases all over the world [2,3]. Sponges are widespread in tropical reefs in a great abundance, but can also be found in polar latitudes and the deep sea as well as in fresh water lakes and rivers [3,4]. Members belonging to the genus *Hyrtios* are classified within Kingdom: Animalia, phylum: Porifera, class: Demospongiae, order: Dictyocerat-ida, and family: Thorectidae [3,5]. The marine sponge *Hyrtios* erectus considered a rich reservoir of pharmacologically active metabolites [6,7]. Various phytochemical com-pounds from genus *Hyrtios* were previously tested against several cell lines such as Dipuupehedione, which exhibited a potent cytotoxic activity on KB cells (IC50 value of 3 μg/mL) [6]. Hyrtinadine A showed in vitro cytotoxic activity with IC50 1 μg/mL against murine leukemia l210 cells [8]. Moreover, sesterstatins 4,5 inhibited the growth of a number of human cancer cell lines, including P388 leukemia, BXPC-3 pancreas, RPMI-7951 melanoma, U251 CNS, KAT-4 thyroid, NCI-H460 lung NSC, FADU pharynx, and DU-145 prostate, with GI50 values (GI50, concentration causing 50% of growth inhibition of cell proliferation) ranging from 1.6 to 4.9 µg/mL [3,9]. Additionally, sesterstatin 6 exhibited significant anticancer activity against murine P388 lymphocytic leukemia (ED50, effective dose causing 50% of the desired activity, 0.17 µg/mL) and a series of human tumor cell lines (GI50 0.18–89 µg/mL) [3,10]. Furthermore, natural quinazoline derivative (2-chloro-6-phenyl-8H-quinazolino[4,3-b]quinazolin-8-one) isolated from marine sponge *Hyrtios* erectus against human breast cancer through inhibition of MCF-7 cells viability with the IC50 value of 13.04 ± 1.03 µg/mL after 48 h via a mechanism that involves ROS production and either extrinsic or intrinsic apoptosis pathways. Moreover, hyrtiosone A as an example of chromanone derivative was furthermore analyzed for in vitro anticancer activity in hepatocellular carcinoma HepG2 cells. Hyrtiosone A demonstrated cell cycle arrest at S and G2/M phase, and resulted in elevated p53 and p27 gene expressions, which signified the chromanone derivative as a prospective therapeutic lead to attenuate hepatocellular carcinoma [11].

**Figure 1:**
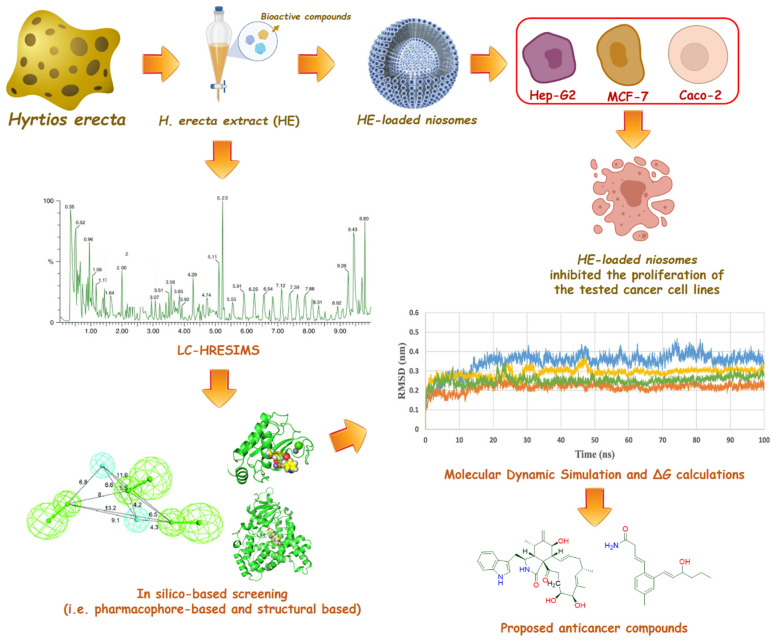
The workflow of this study.

This provoked us to test the ability of the total extract against the three cancer cell lines. Moreover, HR-LCHRMS analysis was carried out for the crude extract to putatively identify its major compounds. Subsequently, these compounds were subjected to a number of in-depth in silico investigation to highlight the most probable anticancer compounds. Although marine products are pharmacologically treasured, their optimum use has been hindered due to the high fragile nature of their commodities and rapid deterioration [12]. Therefore, the application of nanotechnology to deliver and target such important compounds is of high interest. Niosomes are vesicular nano-structures that consist of non-ionic surfactants. They are more advantageous than other nanocarriers, such as liposomes, because they are more stable and cost-effective [13]. Moreover, they are biodegradable, biocompatible, flexible in structure, and guarantee the encapsulation of both hydrophobic and hydrophilic active components [14]. These features encourage the loading of HE in niosomes to investigate if its anticancer potential will be enhanced. The findings in this study could be a starting point towards further investigation of HE and its niosomes as a promising source of anticancer agents. The workflow of this study is depicted in Figure 1.

## 2. Results and Discussion

### 2.1. LC-HRMS Chemical Profiling

**Figure 2:**
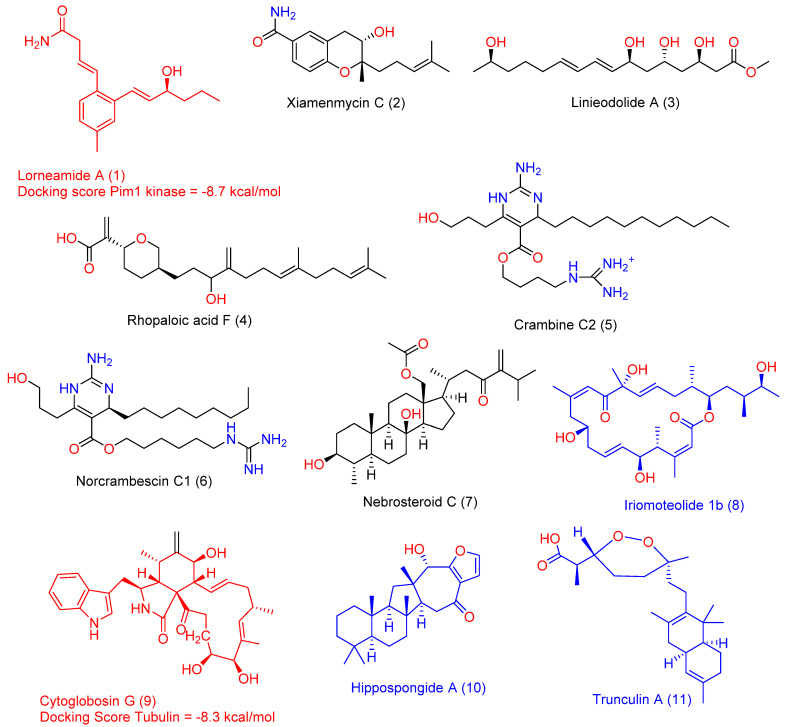
Compounds annotated in HE. Colored structures (**1**, **8**–**11**) can probably act as anti-cancer agents in vitro (Pa > 0.5), while uncolored ones probably cannot. Red-colored structures (compounds **1** and **9**) received significant binding affinity scores (−8.7 and −8.3 kcal/mol, respectively) with two cancer related proteins, human Pim1 kinase and human tubulin.

Metabolic profiling (Figure S1) using HR-LCMS for the sponge *Hyrtios erectus* ex-tract was performed in order to identify chemical compounds responsible for its cyto-toxic activity. Dereplicated compounds, as mentioned in Figure 2, are characterized by being of a different chemical nature. The dereplicated compounds include lorneamide A (**1**) [15], xiamenmycin C (**2**) [16], linieodolide A (**3**) [17], rhopaloic acid F (**4**) [18], crambine C2 (**5**) [19], norcrambescin C1 (**6**) [20], nebrosteroid C (**7**) [21], iriomoteolide 1b (**8**) [22], cytoglobosin G (**9**) [23], hippospongide A (**10**) [24], and trunculin A (**11**) [25] (Table S2).

### 2.2. In Silico-Based Determination of the Active Metabolites

Biological activity predictions with the aid of computers have become a crucial preliminary step in the drug discovery workflow. Such in silico-based experiments are very valuable for exploring natural products for new bioactive metabolite [26]. Accordingly, the structures of the annotated compounds in HE were submitted to the neural network-based prediction software PASS to putatively highlight compounds that might be responsible for the observed anticancer activity of HE. The search proto-col of this software is based on the structural similarity of a great number of inhibitors recorded for a large area of molecular targets [27].

**Figure 3:**
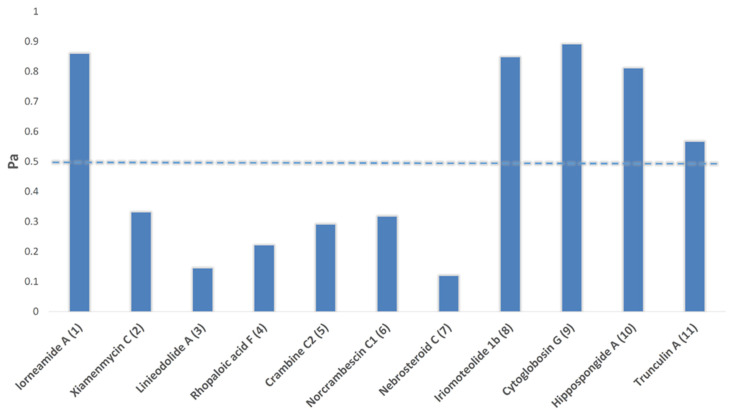
PASS prediction results. Compounds with Pa scores > 0.5 are likely active as anticancer agents in vitro, while those with Pa scores < 0.5 are likely inactive.

As explained in Figure 3, between the annotated compounds in HE, compounds 1 and **8**−**11** were suggested to act as antineoplastic agents (i.e., Pa > 0.5). Previously, compound **8** and **11** have been considered as a potent anticancer agents [28,29].

To support the previous preliminary pharmacophore-based virtual screening step and to obtain further insight into the most probable molecular targets of the predicted anticancer compounds (i.e., compounds **1**, **8**−**11**) were subjected to an inverse docking-based screening using the idTarget inverse docking platform (http://idtarget.rcas.sinica.edu.tw, accessed on 20 July 2022). This software utilizes a docking protocol, divide-and-conquer, which builds small overlapping grids in order to confine the searching space on the protein surfaces, allowing it to perform a large number of precise dockings in a short time. The query structure can be docked against almost all proteins structures hosted in the Protein Data Bank (PDB) (https://www.rcsb.org/, accessed on 20 July 2022). The retrieved results were listed as scores from the highest negative values to the lowest one. To identify the best protein targets for each query structure in HE, with binding affinity of 7 kcal/mol as a cut-off score. As key proteins in the cancer pathogenesis and progression, Pim1 kinase [30] and human tubulin [31] were retrieved as target proteins for compounds **1** and **9** with binding affinity scores of −8.7 and −8.3 kcal/mol, respectively. 

**Figure 4:**
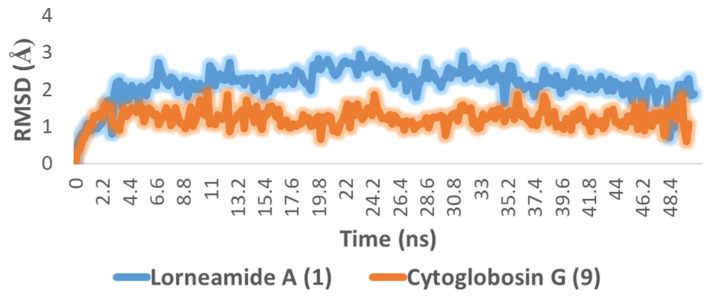
RMSDs of compounds **1** and **9** inside the active sites of both Pim 1 kinase and tubulin, respectively, over 50 ns of MDS.

To further validate the docking results of the previous step, we subjected the re-trieved docking pose of each compound to 50 ns MDS experiments. As depicted in Figure 4, both compounds (i.e., **1** and **9**) were able to achieve stable bindings over the course of simulation producing low binding deviations in comparison with the initial binding modes (i.e., the docking poses) with average RMSDs of 2.1 and 1.1 Å, respectively. Accordingly, their estimated ΔG were found to be considerably low (i.e., ΔG = −8.4 and −7.6, respectively) indicating good affinity toward each corresponding protein target.

**Figure 5:**
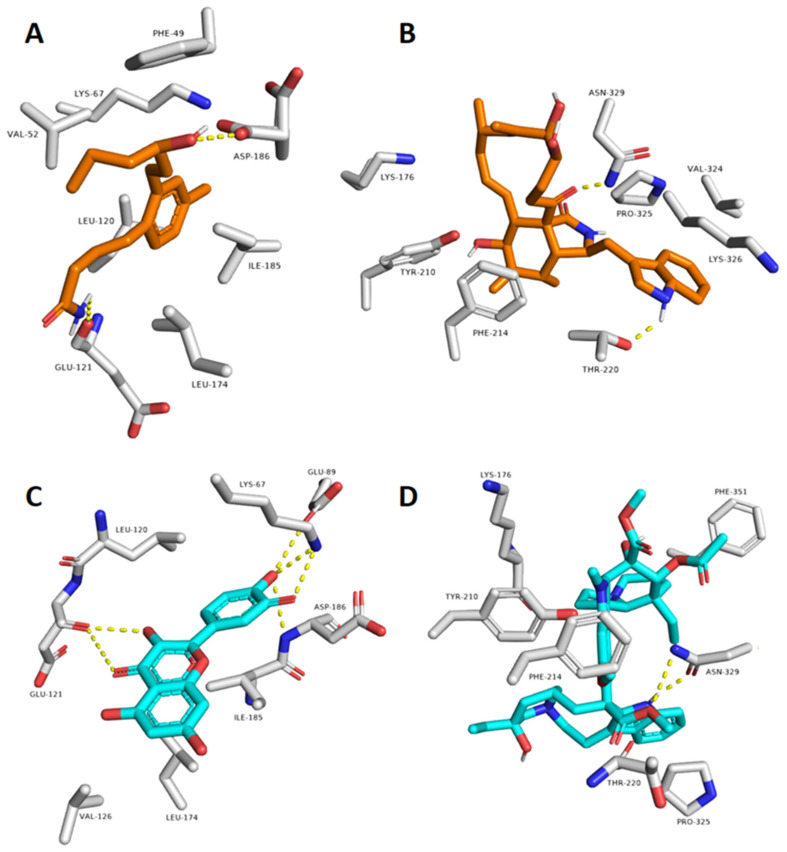
Binding modes of compounds 1 and 9 inside the active sites of both Pim 1 kinase and tubulin extracted form the last snapshots of their MDS (**A**,**B**). (**C**,**D**) are the binding modes of the reference inhibitors co-crystalized with both Pim 1 and tubulin, respectively.

Looking into the binding modes of each compound inside the binding sites of the corresponding proteins at the end of MDS revealed that they were achieved their stable bindings by establishing a number of strong H-bonds (<2.5 Å) and hydrophobic interactions. As shown in Figure 5, compound 1 interacted with GLU-121 and ASP-186 via to hydrogen bonds similarly to the co-crystalized inhibitor, in addition to a number of hydrophobic interactions with LYS-67, LEU-120, LEU-174, and ILE-185. In regard to compound **9**, it established two H-bonds with THR-220 and ASN-329 similarly to the co-crystalized ligand, and was also able to interact with PHE-214, LYS-326, and PHE-351. According to the previous in silico and structural findings, both compounds **1** and **9** are likely linked to the anticancer properties of HE.

### 2.3. HE-Containing Niosomes

**Figure 6:**
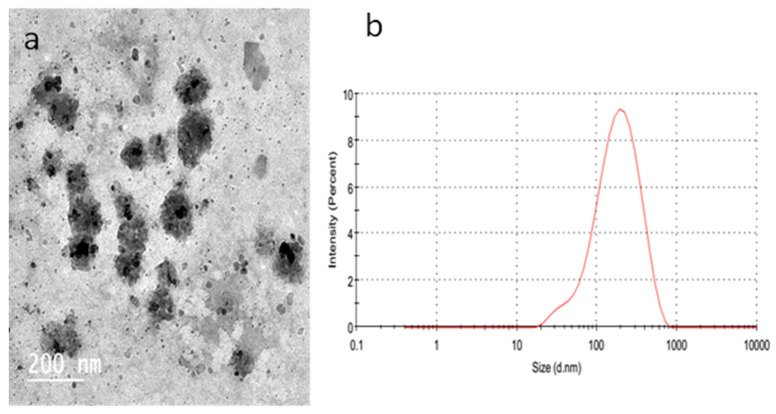
(**a**) TEM of *Hyrtios*-containing niosomes. (**b**) Size distribution of *Hyrtios*-containing niosomes.

**Figure 7:**
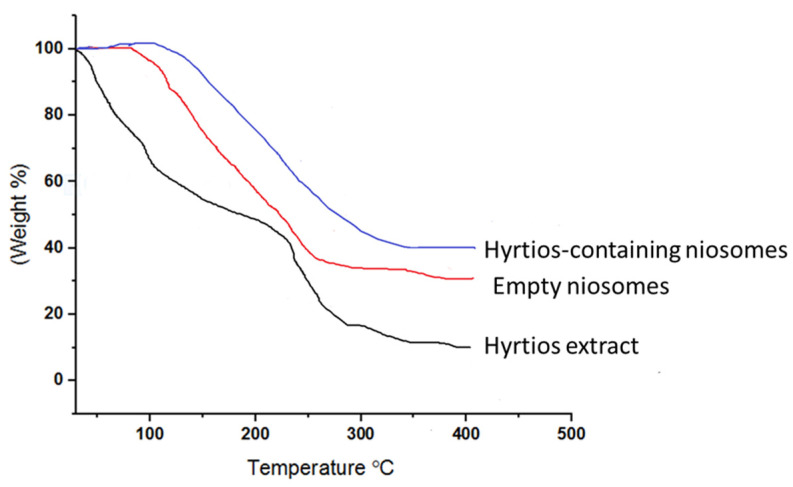
Thermogravimetric analysis declares the enhanced thermal stability of *Hyrtios*-containing niosomes compared to *Hyrtios* extract.

Niosomal formulation of *Hyrtios* erectus extract was successfully prepared as re-vealed by TEM image (Figure 6a). Small vesicular formulation is well developed with homogenous distribution of the formed vesicles (size = 142.3 ± 10.3, PDI = 0.279) (Figure 6b). Prepared niosomes possess a zeta potential of 22.8 ± 1.6. To determine the effect of the prepared niosomes on the promotion of the thermal stability of the encapsulated *Hyrtios* extract as a function of temperature, anocargravimetric study was carried out. TGA curves for empty niosomes, *Hyrtios* extract and *Hyrtios*-containing niosomes are described in Figure 7. Heating with range 30 to 400 °C has resulted in about 52.5 and 22.1% loss of weight at a temperature of 200 °C for *Hyrtios* extract and Hyrtios-containing niosomes, respectively. Results reveal the potential of niosomal encapsulation on the improvement of the thermal stability of the encapsulated *Hyrtios* extract.

**Figure 8:**
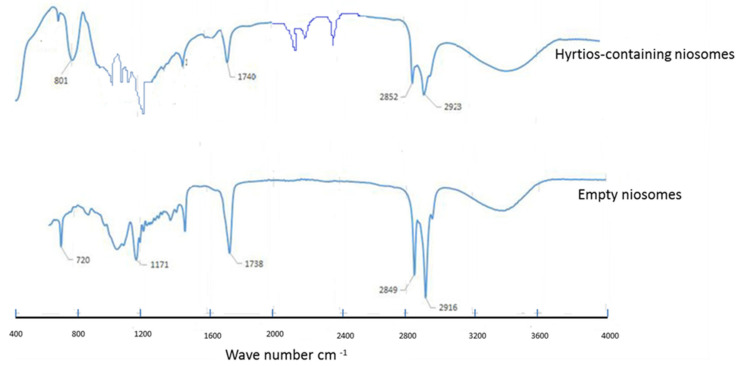
FTIR of empty niosomes and *Hyrtios*-containing niosomes shows no interaction between niosomal components and HE components.

To gain more insight into the possible interactions between the components of both the *Hyrtios* extract and formulated niosomes, FTIR spectra of empty niosomes, and *Hyrtios*-containing niosomes were evaluated (Figure 8). The FTIR spectra show similar bands of empty niosomes and *Hyrtios*- containing niosomes at 2916, 2849, 1738, 1171, and 720 and 2973, 2852, 1740, and 801, respectively. That indicates no developed linkages or interaction have been produced upon encapsulation.

### 2.4. Antiproliferative Potential of the HE and Its Niosomal Formulation

**Figure 9:**
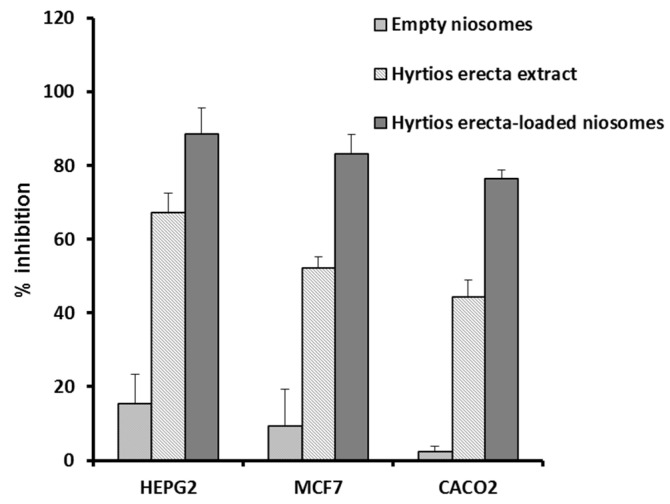
Enhanced inhibition of cellular proliferation of HepG2, MCF7, and Caco-2 cell lines at IC_50_ *HE* niosomes compared to the corresponding empty niosomes and of *HE* extract.

In silico analysis results confirmed, the powerful anticancer potential of HE. Sub-sequently, in vitro investigation for its anti-proliferative activity was performed against the three-studied cells (HepG2, MCF-7, and Caco-2). *Hyrtios erectus* extract showed moderate in vitro anti-proliferative activity towards the studied cell lines with IC50 values 18.5 ± 0.08, 15.2 ± 0.11, and 13.4 ± 0.12, respectively. Doxorubicin was used as a positive control (IC50 4.2, 3.7, 3.4 μg/mL, respectively). Furthermore, we investigated the influence of entrapment in the niosomal formulation on the enhancement of HE extract’s antiproliferative activity. Therefore, MTT assay was carried out for the ex-tract-containing niosomes with the IC50 determination against the three tested cell lines. The formulated HE niosomes (size 142.3 ± 10.3 nm, PDI 0.279, and zeta potential 22.8 ± 1.6) significantly enhanced the in vitro anti-proliferative activity of the encapsulated HE extract (IC50 8.5 ± 0.04, 4.1 ± 0.07, and 3.4 ± 0.05, respectively). This could be at-tributed to the small particle size of the prepared niosomes which promotes cellular in-take and internalization. This is incontinence with previous results that identified niosomes as potential delivery systems in anticancer therapy. Moreover, recent study has revealed the impact of niosomal formulation in preferential targeting of the uploaded cargo to the tumor tissues [32]. Evaluation of anti-proliferative potential, as shown in Figure 9, for the three tested cell lines of HE extract, HE niosomes, and empty niosomes as well as to eliminate phospholipid membrane cytotoxic effect. The results come in match with that reported which reflected the impact of nanocarriers on the improvement of the cellular uptake and availability of the encapsulated substances [33].

### 2.5. Network Pharmacology

#### 2.5.1. Networks

##### Sponge–Metabolite Network

Based on chemical analysis of Red sea sponge *H. erectus* using HR-LCMS, metabolic profiling was identified. The identified 11 metabolites were connected to the sponge extract.

##### The Metabolites–Target Genes Network

**Figure 10:**
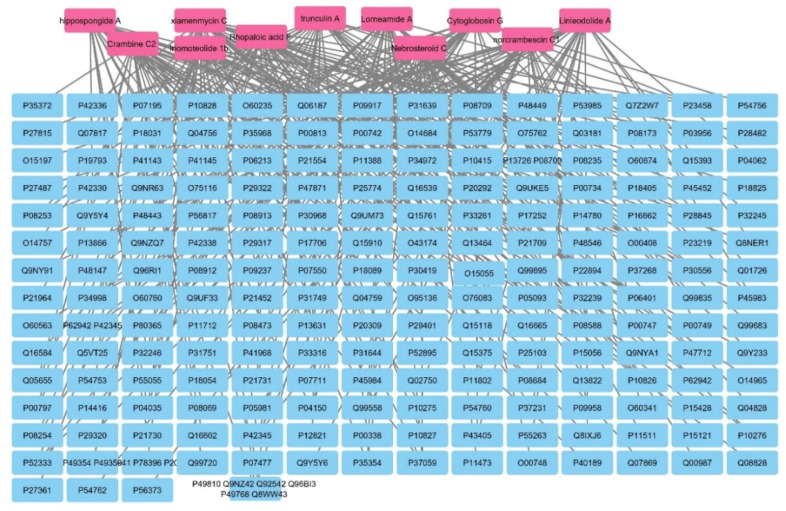
Metabolites–all targets network. The pink rectangles represent the active metabolites of *Hyrtios erectus* sponge extract; the blue rectangles represent all genes (genes are mentioned in Uniprot IDs).

The target genes related to the identified metabolites were predicted by BindingDB database and Swiss Target Prediction database. The active metabolites were connected to 200 genes as illustrated in Figure 10.

##### Targets–Cancer Types (Breast, Colon, and Hepatocellular) Network

Target genes were connected to different classes of three cancer types (breast, colon, and liver cancers) using the DisGenet online database and FunRich software. This result in 147 genes among the resulting gene list related to the three types of cancer( breast, colon, and liver) in different classes. The network of the target genes—(breast cancer, colon cancer, and liver cancer)—is composed of 150 nodes and 333 edges, with a characteristic path length of 2.004 and network centralization of 0.929.

**Figure 11:**
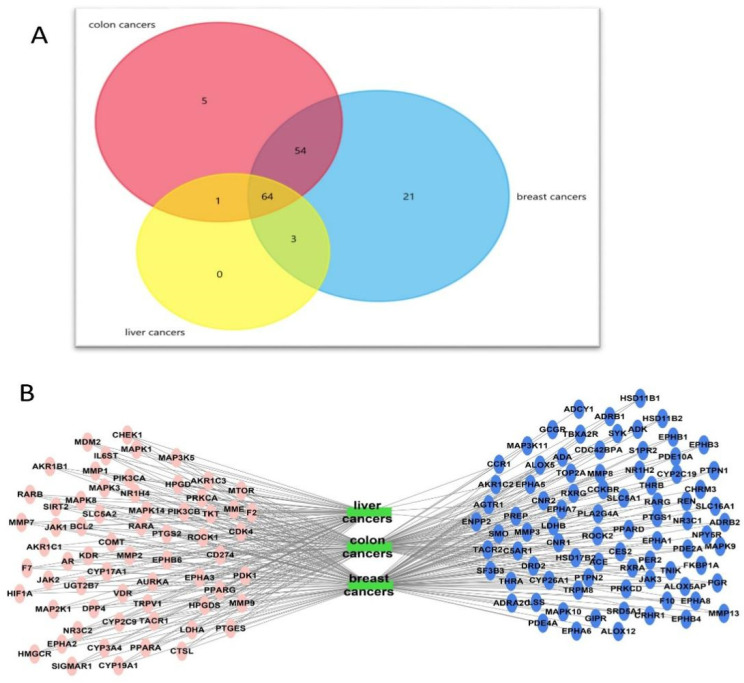
(**A**) A Venn diagram illustrating the number of intersected genes involved in the three cancer types, breast, colon, and liver, among the identified targets. (**B**) A network of target genes- major cancers (breast, colon, and liver). Green rectangles represent types of cancers; oval pink shapes represent the intersected genes between the three types of cancers; and oval blue shapes represent genes not involved in the three types of cancers.

There are 147 nodes represented by the target genes and 3 nodes represent the three cancer types of liver, colon, and breast; the intersected genes (present in the three cancer types: colon, breast, and liver) are 64 genes as illustrated in Figure 11A,B.

##### Sponge–Metabolite–Intersected Cancer Genes

**Figure 12:**
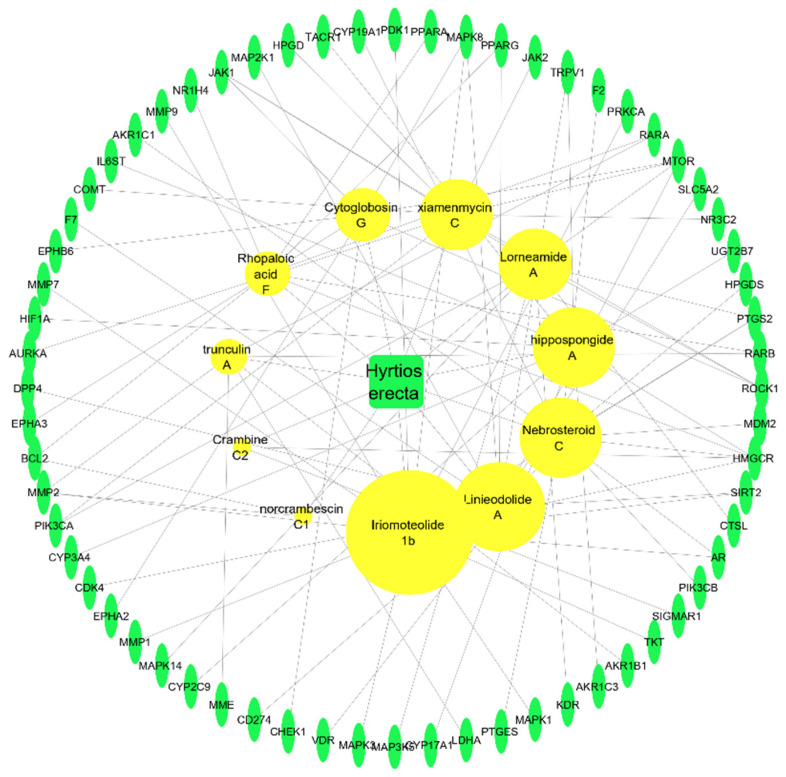
Sponge–metabolite–intersected cancer genes network, which is a network between the active metabolites of *Hyrtios erectus* and the intersected genes. Yellow circles represent the active metabolites (the larger the circle, the more edges); the green oval shapes represent the intersected genes among the three types of cancer.

The network of the sponge–metabolites–intersected genes related to the major studied genes of breast, colon, and hepatocellular cancers consists of 76 nodes (one for the sponge *H. erectus),* 11 nodes corresponding to the active metabolites, and 64 nodes represent the intersected genes to the studied cancer types. The network contains 100 edges representing the number of interactions of each compound and intersected genes among our gene set, as illustrated in Figure B. The network showed that compound iriomoteolide 1b has the top number of edges at 14 edges, followed by nebrosteroid C with 11 edges, then hippospongide A and linieodolide A with 10 edges each, and finally lorneamide and xiamenmycin with 9 edges each as illustrated in Figure 12.

##### Sponge–Metabolites–Intersected Genes–Classes of Types of Cancer

**Figure 13:**
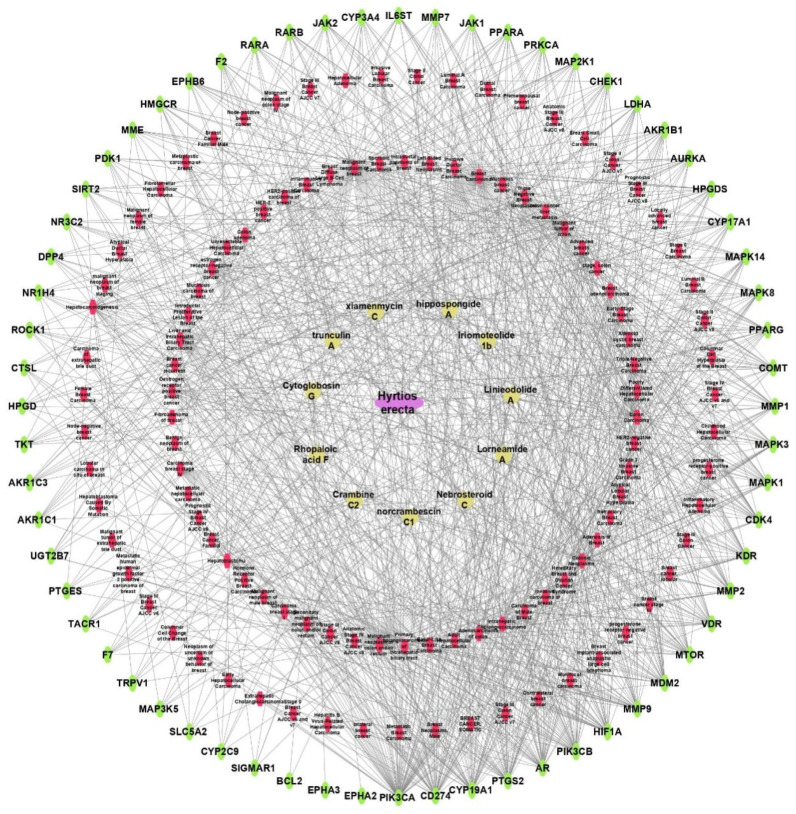
Sponge–metabolites–intersected genes–classes of cancers related to breast, colon, and hepatocellular cancers. The oval pink node represents the sponge’s name; yellow circles represent the active metabolites; green oval shapes represent the target intersected genes; and the red oval shapes represent different classes of breast, colon, and hepatocellular cancers related to the target set of genes.

The complete network was constructed between the sponge, the active metabolites, and the target genes that have influence on the three main types of cancer: colon cancer, breast cancer, and hepatocellular carcinoma. These genes are related to different classes of the three mentioned types of cancers. The network consisted of 189 nodes: 11 nodes for the metabolites, 64 nodes for the intersected genes, 1 node for the sponge name, and 113 nodes for the different classes of breast, colon, and hepatocellular cancers as illustrated in Figure 13

#### 2.5.2. Gene Ontology and Enrichment Analysis

**Figure 14:**
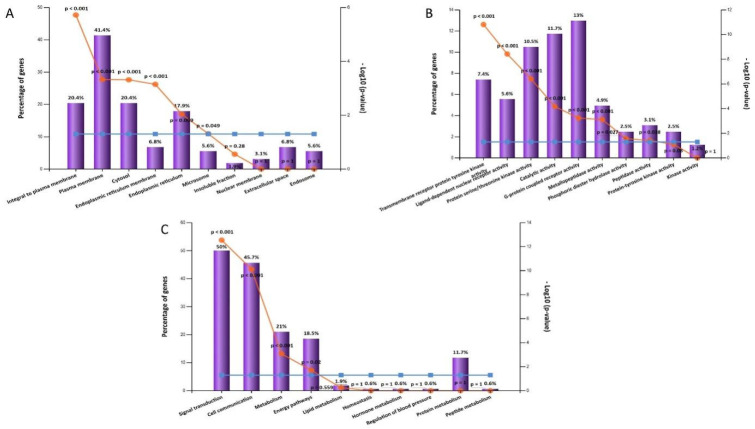
Gene enrichment analysis of target genes of *H. erectus* active metabolites showing (**A**) cellular components, (**B**) molecular functions, and (**C**) biological processes.

**Figure 15:**
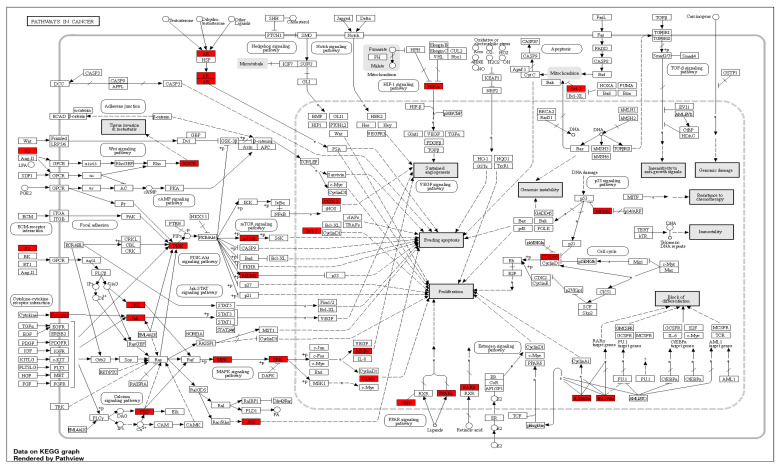
KEGG illustrating diagram of biological pathways showing the pathway in cancer as the top pathway and evolved direct and indirect genes (AR, BCL-2, CDK4, COX-2, CDK 4/6, ERK, F2, HIF-α, HZFRAR-α, HMRAR-α, JNK, JAC, MEK, MMPs, ROCK, P13K, RARβ, PKC, PPFP, PPARy).

The gene ontology and enrichment analysis was performed on all targets of the active metabolites to discover the cellular components, molecular function, and biological processes that were affected by this set of genes using FunRich software. The analysis showed that cellular components are plasma membrane, cytosol, and integral to plasma membrane. These are the top cellular components in the same order (Figure 14A). G-protein coupled receptor activity was the top molecular function followed by catalytic activity and protein serine/threonine kinase activity (Figure 14B). The top biological processes were signal transduction followed by cell communication and metabolism (Figure 14C). ShinyGO v0.741 enrichment analysis was used to discover the top biological pathways related to the target genes according to represented number of genes (Table S1, Figure 15).

## 3. Material and Methods

### 3.1. Extraction of Sponge Material 

The marine sponge *Hyrtios erectus* was obtained from Sharm El- Shaikh, South Sinai Governorate, Egypt. The sponge was identified by Prof. El-Sayd Abed El-Aziz (Department of Invertebrates Lab., National Institute of Oceanography and Fisheries, Red Sea Branch, 84511 Hurghada, Egypt). The fresh sponge *Hyrtios erectus* (100 g) was collected, freeze dried, and then reduced to fine powder (40 g). The powdered sponge was then macerated in MeOH: DCM (1:1) to create 6 g of the total extract.

### 3.2. LC-HRMS Analysis

LCMS was performed using a Synapt G2 HDMS quadrupole time-of-flight hybrid mass spectrometer (Waters, Milford, CT, USA). BEH C18 column was adjusted to 40 °C then connected to the guard column to be ready to inject the sample (2 μL). The mobile phase with gradient elution was used. Mzmine 2.12 was maintained for differential analysis of MS data. ProteoWizard was employed for convention of the raw data into positive and negative files in mzML format.

### 3.3. Preparation of Niosomes of Hyrtios Erectus Extract

Niosomes were prepared by adopting the spraying technique reported previously [34]. Briefly, certain amounts of Span 60 and cholesterol (2:1 M ratio) and 5 mg *Hyrtios* extract were dissolved in 2.5 mL ethanol (absolute) forming the organic phase. The organic phase was loaded in a closed spraying device. The organic phase was then finely sprayed in a closed system containing 2 mL 9% *w/v* sucrose solution (2 mL per 10 s). The whole system was heated to 60 °C and continuously stirred at 1200 rpm. Stirring at 1200 rpm was continued for 20 min until the complete evaporation of ethanol was reached and spontaneous formation of niosomes occurred. The formulated niosomal suspension was kept for 24 h at 4 °C to complete the strengthening of the formed bilayer. Prepared niosomes were stored in the refrigerator for further investigations. 

#### 3.3.1. Transmission Electron Microscopy (TEM)

Diluted samples of the prepared niosomal suspension of *Hyrtios* extract (1:150 *v/v*) were visualized using (JEM-1400, Jeol, Tokyo, Japan) adjusted at 80-kV. Niosomes were dried at 40 °C on copper grids left for 15 minutes before imaging [35].

#### 3.3.2. Size of HE-Containing Niosomes

Size and poly-dispersity index of prepared niosomes were evaluated with a Zetasizer-Nano-ZSP (Malvern-Instruments, Malvern, UK). Dynamic light scattering and electrophoretic mobility of the diluted samples (1:150 *v/v*) were used to determine the hydrodynamic diameter and zeta potential, respectively.

#### 3.3.3. FTIR and TGA of HE-Containing Niosomes

For further estimation of the stability of the prepared formulation, the change in the weight with changing temperature of free *Hyrtios erectus* extract, empty niosomes, and HE-containing niosomes was determined by using thermogravimetric analysis (TGA). Dried samples were heated from 30 to 400 ºC using a heat flow rate of 20 °C/min and nitrogen (flow rate 20 mL/min). Moreover, the possible interaction among Span 60, cholesterol, and *Hyrtios* extract was studied using Fourier-transform infrared (FT-IR) spectroscopy for the empty niosomes and *Hyrtios erectus* extract-containing niosomes along a range of wavenumber from 4000 to 400 cm (Nicollet IS 10 FTIR spectrometer, Thermo fisher, Maidson, WI, USA) following dispersing the samples in discs of KBr. 

### 3.4. In Vitro MTT Assay

#### 3.4.1. Conditions of Cell Culture 

HepG2, CACO_2_, and MCF7 cell lines were kept separately in RPMI-1640 (Gibco® by Life Technologies, Grand Island, NY, USA), which were enhanced with fetal bovine serum (FBS) (10%) (Gemini Bio-Products, Sacramento, CA, USA), 100 mL U/mL penicillin G, 100 μM/mL streptomycin, and 2 mmol/L glutamine. Cells were cultured in DMEM (Invitrogen, Life Technologies) and enhanced with FBS (Hyclone) (10%), insulin (10 μg/mL) (Sigma), and penicillin-streptomycin 1%.

#### 3.4.2. Antiproliferative Potential of *HE* and *HE*-Containing Niosomes

Breast cell lines (MCF-7), liver cell lines (HepG-2), and colorectal cell lines (Caco-2) were maintained in earth RPMI 1640 medium with heat-inactivated FBS (10%) then grown in 5% CO_2_ at 37 °C. Then, 96-well cell culture plates were used to seed the tested cell lines (2 × 10^4^ cells/well in the case of MCF-7 and HepG-2 and 6 × 10^4^ cells/well in the case of Caco-2 cells). The anti-proliferative potential of the empty niosomes and HE—either free or encapsulated within the niosomal formulation—was evaluated. Concentrations of 0, 5, 12.5, 25, and 50 µg/mL of the respective extract were included. Cell viability was determined using 3-(4,5-dimethylthiazol-2-yl)-2,5-diphenyltetrazolium bromide (MTT) assay 8. Plates including triplicate of untreated cells were considered as 100% viability. Plates with triplicates of cells managed by cytotoxic reagents (200 ng/mL TNF, 200 ng/mL TRAIL, 200 ng/mL CD95L, 5 μg/mL CHX, and 1% (*w/v*) sodium azide 20%) were supposed as 0% viability. Average of triplicates were analyzed using Graph Pad Prism 5 software (La Jolla, CA, USA).

### 3.5. Molecular Modeling

#### 3.5.1. *In Silico* Biological Activity Predictions

PASS software [27] was employed for the prediction of the most possible anticancer compounds among the annotated metabolites in HE. The details of PASS-based predictions are described in the Supplementary Material and Methods.

#### 3.5.2. Prediction of the Potential Protein Targets

The highly probable anticancer compounds predicted in the previous neural networking pharmacophore-based screening were subjected to an inverse docking-based virtual screening using the idTarget platform (http://idtarget.rcas.sinica.edu.tw/, accessed on 22 July 2022) [36,37,38]. The details of idTarget-based screening are discussed in the Supplementary Material and Methods.

### 3.6. Network Pharmacology Study

#### 3.6.1. Networks Construction

##### Sponge–Metabolite Network

Based on chemical analysis of Red Sea sponge *H. erectus* using HR-LCMS, metabolic profiling was identified. The identified 11 metabolites were connected to the sponge extract in a simple network.

##### The Metabolites–Target Genes Network

The target genes related to the identified metabolites were predicted by PubChem (https://pubchem.ncbi.nlm.nih.gov/, accessed on 20 July 2022) [39], Binding DB (https://www.bindingdb.org/rwd/bind/index.jsp, accessed on 20 July 2022) [40] and Sawiss Target Prediction (http://www.swisstargetprediction.ch/, accessed on 20 July 2022) [41] database. The top target genes were selected based on a similarity index of more than 0.7 in Binding DB and we chose the top targets in Swiss Target Prediction database [41] using canonical smiles for each structure as input data method, the human species (*Homo sapiens*) was selected.

##### Targets–Cancer Types (Breast, Colon, and Hepatocellular)

The DisGenet (https://www.disgenet.org/, accessed on 20 July 2022) [42] online database was used to discover the targets for specific cancers by using specific filter words: ‘cancer’, ‘melanoma’, and ‘carcinoma’. Then, filtration of the downloaded DisGenet results was conducted to specify the cancer results to words (liver, hepatocellular, colon, and breast). 

##### Sponge–Metabolite–Intersected Cancer Genes

The network of the sponge–metabolites–intersected genes related to the major studied genes of breast, colon, and hepatocellular cancers.

##### Sponge–Metabolites–Intersected Genes–Classes of Types of Cancer

The complete network was constructed between the sponge and the active metabolites and the target genes that have influence on the three main types of cancer: colon cancer, breast cancer and hepatocellular carcinoma. The networks visualization was performed using the software Cytoscape 3.9.0. (https://cytoscape.org/download.html, accessed on 8 August 2022) [43].

#### 3.6.2. Gene ontology and Enrichment Analysis

The gene ontology and enrichment analysis was performed on all the targets of the active metabolites to discover the cellular components, molecular function, and biological processes that are affected by this set of genes using FunRich version 3.1.3. (http://www.funrich.org, accessed on 10 August 2022) [44]. The enrichment analysis was performed using the KEGG database (https://www.genome.jp/kegg/, accessed on 12 August 2022) [45] and ShinyGo database 0.76.1 (http://bioinformatics.sdstate.edu/go/, accessed on 13 August 2022, a graphical gene set enrichment tool) [46].

## 4. Conclusions

Herein, the LC-HRMS-assisted chemical profiling of Red Sea *Hyrtios erectus* (HE) led to the annotation of 11 compounds of different chemical classes, e.g., polyketides, terpenoids, steroids, and indole alkaloids. Upon in vitro anticancer testing, HE showed a moderate antiproliferative effect against the MCF-7, HepG2, and Caco-2 cell lines; IC_50_ ranged from 13–18 µg/mL. The improved uptake by the studied cell lines was obtained by the encapsulation of HE within niosomal formulation. The results reveal the impact of entrapment of the HE extract as a promising approach to enhance the anti-proliferative potential of the extract. However, further in vivo studies should be implemented to confirm that concept. PASS software-assisted virtual screening highlighted a number of annotated compounds (i.e., compounds **1** and **8**–**11**) that are associated with the anticancer potential of HE compounds. This study also confirmed the preferability of niosomes as a nanocarrier for improvement of the anticancer activity of the HE extract. The network pharmacology analysis that focused on target genes involved in the three types of studied cancer cell lines (breast, colon, and liver) revealed that compounds **1**, **2**, **3**, **7**, **8**, and **10** are the major compounds responsible for the anticancer potential of the three cell lines and that the pathways in cancer are the top pathways of the target genes. The docking study results and pharmacology networking study revealed that compounds **1**, **8**, and **10** have a high binging score and contain more anticancer genes so the co-occurrence of Lorneamide A, Iriomoteolide 1b, and hippospongide A in the HE extract might be responsible for the anticancer potential of the extract.

## Supplementary Materials

The following are available online at https://www.mdpi.com/article/10.3390/md20100628/s1, Figure S1: Total ion chromatogram of crude extract of Hyrtios erectus, Table S1: KEGG biological pathway analysis of target genes related to Hyrtios erecta extract, the pathways are arranged in descending order according to enrichment FDR and number of genes, Table S2: Compounds annotated in *Hyrtios erectus* extract.
