# Supplementary material for: Network Pharmacological Analysis of the Red Sea Sponge Hyrtios erectus Extract to Reveal Anticancer Efficacy of Corresponding Loaded Niosomes
Source: Mar Drugs. 2022 Oct 1;20(10):628. doi: 10.3390/md20100628 (PMC9604794; doi:10.3390/md20100628)
Supplement: Supplementary file 1 [file marinedrugs-20-00628-s001.zip › marinedrugs-1913162-supplementary.pdf]

Network Pharmacological Analysis of the Red Sea Sponge Hyrtios erectus Extract to Reveal Anticancer Efficacy of Corresponding Loaded Niosomes

Heba A. Abou-Taleb [1,†], Ahmed M. Sayed [2,3,†], Hesham Refaat [4,5], Faisal Alsenani [6], Eman Alaaeldin[4,5], Fatma A. Mokhtar [7], Usama Ramadan Abdelmohsen [8,9,*] and Nourhan Hisham Shady [9,*]

[1] Department of Pharmaceutics and Industrial Pharmacy, Merit University (MUE), New Sohag 82755, Egypt
[2] Department of Pharmacognosy, Faculty of Pharmacy, Al-Maaqal University, Basra 61014, Iraq
[3] Department of Pharmacognosy, Faculty of Pharmacy, Nahda University, Beni-Suef 62513, Egypt
[4] Department of Pharmaceutics, Faculty of Pharmacy, Minia University, Minia 61519, Egypt
[5] Department of Pharmaceutics, Faculty of Pharmacy, Deraya University, 7 Universities Zone, New Minia 61111, Egypt
[6] Department of Pharmacognosy, College of Pharmacy, Umm Al-Qura University, Makkah 21955, Saudi Arabia
[7] Department of Pharmacognosy, Faculty of Pharmacy, Alsalam University, Kafr El Zayat 31616, Egypt
[8] Department of Pharmacognosy, Faculty of Pharmacy, Minia University, Minia 61519, Egypt
[9] Department of Pharmacognosy, Faculty of Pharmacy, Deraya University, Universities Zone, Minia 61111, Egypt
† These authors have equally contributed to this work
* Correspondence: usama.ramadan@mu.edu.eg (U.R.A.), Norhan.shady@deraya.edu.eg.(N.H.S.);
Tel.: +2-86-2347759 (U.R.A.); Fax: +2-86-2369075 (U.R.A.)

Material and Methods

In Silico Biological Activity Predictions

PASS [1] was employed for the prediction of the most possible anticancer metabolites in *Hyrtios erecta.*, and to point a probable molecular target for them. This software was capable of to predict >4000 types of pharmacological and toxicological activities including their mechanism of action, with approximately 85% as acceptable precision, depending on the submitted compound structures that were subsequently screened applying the structure activity relationship database (SARBase). The prediction results were given as probability scores (probably active "Pa" or probably inactive "Pi").

These calculated probability scores were determined by linking the structure and functional groups features in the tested molecules that matched or mismatched the specific activities recorded in the software associated database. The higher the Pa values, the better acceptable it was for the compound to present the suggested pharmacological activity on a scale of 0–1. Pa values higher than 0.5 mean a high experimental chance of the suggested pharmacological activity.

Prediction of the Potential Protein Targets

By performing inverse docking against all proteins in the Protein Data Bank (PDB; https://www.rcsb.org/), potential protein targets for the CE discovered compounds were identified. This task was accomplished with the help of the idTarget platform (http://idtarget.rcas.sinica.edu.tw/). This structural-based screening software uses a unique docking approach known as divide-and-conquer docking, in which it adaptively builds small overlapping grids to constrain the searching space on protein surfaces, allowing it to run a large number of accurate docking experiments in a shorter amount of time. [2] The data were collected as a list of binding affinity scores, organized from the most negative to the least negative. To identify the optimal targets for compounds 1, 8-11 that were predicted as potential anticancer compounds, we used a binding affinity score of -7 kcal/mol as a cut-off number. Accordingly, Pim 1 kinase and human tubulin was selected as a cancer related targets for compounds 1 and 9, respectively.

Molecular Dynamics Simulation

Desmond v. 2.2 software was used for performing MDS experiments .[3-5] This software applies the OPLS-2005 force field. Protein systems were built using the System Builder option, where the protein structure was checked for any missing hydrogens, the protonation states of the amino acid residues were set (pH = 7.4), and the co-crystalized water molecules were removed. Thereafter, the whole structure was embedded in an orthorhombic box of TIP3P water together with 0.15 M Na+ and Cl− ions in 20 Å solvent buffer. Afterward, the prepared systems were energy minimized and equilibrated for 10 ns. For proteinligand complexes, the top-scoring poses were used as a starting points for simulation. Desmond software automatically parameterizes inputted ligands during the system building step according to the OPLS force field. For simulations performed by NAMD [5], the protein structures were built and optimized by using the QwikMD toolkit of the VMD software. The parameters and topologies of the compounds were calculated either using the Charmm27 force field with the online software Ligand Reader and Modeler (http://www.charmm-gui. org/?doc=input/ligandrm, accessed on 16 April 2021) [6] or using the VMD plugin Force Field Toolkit (ffTK) (compounds 3, 4, 9, 10). Afterward, the generated parameters and topology files were loaded to VMD to readily read the protein–ligand complexes without errors and then conduct the simulation step.

Binding Free Energy Calculations

Binding free energy calculations (ΔG) were performed using the free energy perturbation (FEP) method. [6] This method was described in detail in the recent article by Kim and coworkers.[6]Briefly, this method calculates the binding free energy $\Delta G_{binding}$ according to the following equation: $\Delta G_{binding} = \Delta G_{Complex} - \Delta G_{Ligand}$. The value of each ΔG is estimated from a separate simulation using NAMD software. All input files required for simulation by NAMD can be prepared by using the online website Charmm-GUI (https://charmm-gui.org/?doc=input/afes.abinding, accessed on 18 May 2021). Subsequently, we can use these files in NAMD to produce the required simulations using the FEP calculation function in NAMD. The equilibration (5 ns long) was achieved in the NPT ensemble at 300 K and 1 atm (1.01325 bar) with Langevin piston pressure (for "Complex" and "Ligand") in the presence of the TIP3P water model. Then, 10 ns FEP simulations were performed for each compound, and the last 5 ns of the free energy values were measured for the final free energy values .[6] Finally, the generated trajectories were visualized and analyzed using VMD software.

Table S1. KEGG biological pathway analysis of target genes related to *Hyrtios erecta extract*, the pathways are arranged in descending order according to enrichment FDR and number of genes.

Enrichment FDR	nGenes	Pathway Genes	Fold Enrichment	Pathway
3.14E-22	25	530	16.80129717	Pathways in cancer
5.92E-17	13	95	48.74144737	Endocrine resistance

7.70E-16	15	202	26.44956683	Proteoglycans in cancer
9.87E-15	11	79	49.59572785	EGFR tyrosine kinase inhibitor resistance
9.87E-15	14	194	25.70425258	Kaposi sarcoma-associated herpesvirus infection
9.87E-15	14	197	25.31281726	Chemical carcinogenesis
2.68E-14	14	214	23.30198598	Lipid and atherosclerosis
2.93E-14	11	89	44.02317416	PD-L1 expression and PD-1 checkpoint pathway in cancer
9.86E-14	11	100	39.180625	AGE-RAGE signaling pathway in diabetic complications
2.36E-13	11	109	35.94552752	HIF-1 signaling pathway
4.46E-13	12	161	26.54813665	Hepatitis B
4.46E-13	12	161	26.54813665	MicroRNAs in cancer
4.88E-13	11	119	32.92489496	Sphingolipid signaling pathway
7.45E-13	13	224	20.67159598	Human cytomegalovirus infection
9.79E-13	9	59	54.33368644	VEGF signaling pathway
9.79E-13	11	129	30.37257752	Relaxin signaling pathway
1.96E-12	10	97	36.72036082	Prostate cancer
3.97E-12	9	70	45.79553571	Prolactin signaling pathway
3.97E-12	9	70	45.79553571	Central carbon metabolism in cancer
5.01E-12	10	108	32.98032407	Th17 cell differentiation

Table S2. Compounds annotated in *Hyrtios erectus* extract.

No	Compound name	Ionization mode	M/Z	RT	Molecular formula
1	Lorneamide A	P	273.1729	3.7	$C_{17}H_{23}NO_2$
2	Xiamenmycin C	P	289.1678	2.5	$C_{17}H_{23}NO_3$
3	Linieodolide A	P	330.2042	5.0	$C_{17}H_{30}O_6$
4	Rhopaloic acid F	P	390.2770	19.5	$C_{24}H_{38}O_4$
5	Crambine C2	P	467.3709	12.0	$C_{24}H_{47}N_6O_3$

6	Norcrambescin C1	P	466.3631	2.7	$C_{24}H_{46}N_6O_3$
7	Nebrosteroid C	P	502.3658	14.5	$C_{31}H_{50}O_5$
8	Iriomoteolide 1b	P	506.3243	14.4	$C_{29}H_{46}O_7$
9	Cytoglobosin G	P	548.2886	15.3	$C_{32}H_{40}N_2O_6$
10	Hippospongide A	P	384.2664	16.3	$C_{25}H_{36}O_3$
11	Trunculin A	P	390.2770	19.1	$C_{24}H_{38}O_4$

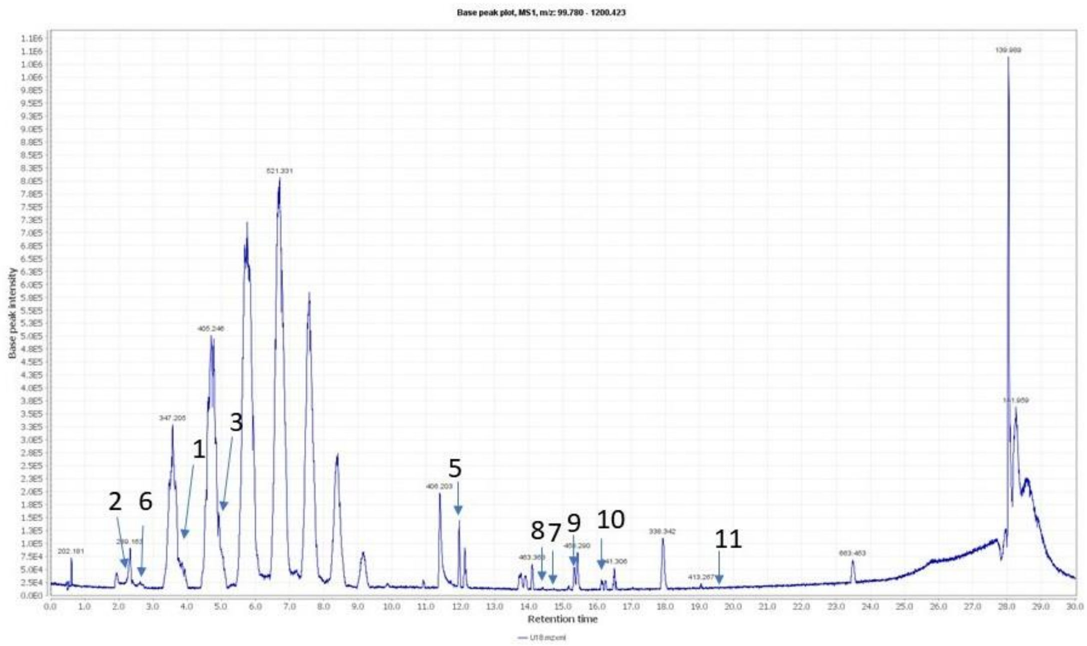

Figure S1. Total ion chromatogram of crude extract of *Hyrtios erectus*.

References

1. Lagunin, A.; Stepanchikova, A.; Filimonov, D.; Poroikov, V. PASS: prediction of activity spectra for biologically active substances. *Bioinformatics* **2000**, *16*, 747-748.
2. Wang, J.-C.; Chu, P.-Y.; Chen, C.-M.; Lin, J.-H. idTarget: a web server for identifying protein targets of small chemical molecules with robust scoring functions and a divide-and-conquer docking approach. *Nucleic acids research* **2012**, *40*, W393-W399.
3. Bowers, K.J.; Chow, D.E.; Xu, H.; Dror, R.O.; Eastwood, M.P.; Gregersen, B.A.; Klepeis, J.L.; Kolossvary, I.; Moraes, M.A.; Sacerdoti, F.D. Scalable algorithms for molecular dynamics simulations on commodity clusters. In Proceedings of the SC'06: Proceedings of the 2006 ACM/IEEE Conference on Supercomputing, 2006; pp. 43-43.
4. Release, S. 3: Desmond molecular dynamics system, DE Shaw research, New York, NY, 2017. *Maestro-Desmond Interoperability Tools, Schrödinger, New York, NY* **2017**.
5. Schrödinger, L. New York, NY, USA, 2012. *LigPrep. version* **2018**, *2*.
6. Kim, S.; Oshima, H.; Zhang, H.; Kern, N.R.; Re, S.; Lee, J.; Roux, B.; Sugita, Y.; Jiang, W.; Im, W. CHARMM-GUI free energy calculator for absolute and relative ligand solvation and binding free energy simulations. *Journal of chemical theory and computation* **2020**, *16*, 7207-7218.
